# Optimal Surgical Extent in Patients with Unilateral Multifocal Papillary Thyroid Carcinoma

**DOI:** 10.3390/cancers14020432

**Published:** 2022-01-15

**Authors:** Joohyun Woo, Hyungju Kwon

**Affiliations:** Department of Surgery, Ewha Womans University Medical Center, Seoul 07985, Korea; jwoo@ewha.ac.kr

**Keywords:** papillary thyroid carcinoma, multifocality, lobectomy, operative extent

## Abstract

**Simple Summary:**

Around 30% of patients with papillary thyroid cancer (PTC) have multifocality. As tumor multifocality could increase the risk of recurrence in patients with PTC, more aggressive treatments, including total thyroidectomy and higher-dose radioiodine, are commonly used to treat patients with multifocal PTC. However, it is unclear whether aggressive treatment can decrease the risk of recurrence. Our study of 718 patients demonstrated that thyroid lobectomy showed comparable recurrence-free survival to that of total thyroidectomy. Moreover, our findings indicated that thyroid lobectomy could be safely performed on multifocal PTC patients with high-risk factors, such as large tumor size or lymph node metastasis. In conclusion, thyroid lobectomy was not associated with the risk of recurrence in patients with multifocal PTCs. Multifocality in PTC may not always require aggressive surgery.

**Abstract:**

Multifocality increases the risk of recurrence in patients with papillary thyroid carcinoma (PTC); however, it is unclear whether multifocality justifies more extensive or aggressive surgical treatment. Here, we evaluated the effect of the operative extent on the recurrence-free survival (RFS) of patients with multifocal PTC. Between 2010 and 2019, 718 patients with unilateral multifocal PTC were enrolled; 115 patients (16.0%) underwent ipsilateral thyroid lobectomy, and 606 patients (84.0%) underwent total thyroidectomy. With a mean follow up of 5.2 years, RFS was comparable between the total thyroidectomy and lobectomy groups (*p* = 0.647) after adjusting for potential confounders. Multivariable Cox regression analysis also demonstrated that the operative extent was not an independent predictor of recurrence (HR 1.686, 95% CI: 0.321–8.852). Subgroup analyses further indicated that both total thyroidectomy and thyroid lobectomy resulted in comparable RFS for multifocal PTC patients with other high-risk factors, including tumor size > 1 cm (*p* = 0.711), lymph node metastasis (*p* = 0.536), and intermediate ATA risk of recurrence (*p* = 0.682). In conclusion, thyroid lobectomy was not associated with the risk of recurrence in patients with multifocal PTCs. Multifocality in PTC may not always require aggressive surgery.

## 1. Introduction

Thyroid cancer is the ninth most prevalent cancer worldwide, and its incidence has dramatically increased over the last four decades [1]. There were 586,202 new cases of thyroid cancer in 2020, and papillary thyroid carcinoma (PTC) represented over 80% of all thyroid cancers [1]. Surgery for thyroid cancer is the most important element of a multifaceted treatment approach [2]. Earlier guidelines recommended total thyroidectomy as the initial surgical treatment option, whereas the latest 2015 American Thyroid Association (ATA) guidelines have endorsed that thyroid lobectomy is safe and sufficient in selected patients with a low to intermediate risk of recurrence [2,3,4]. The optimal surgical extent can be determined by several clinicopathological factors, including personal history of radiation treatment to the head and neck, familial history of thyroid cancer, tumor size, extrathyroidal extension (ETE), regional or distant metastases, and multifocality [2].

Multifocality is defined as the simultaneous presence of two or more tumor foci within the thyroid gland [5]. Tumor multifocality is a common finding in PTC, with a prevalence of 18–87% of cases in the literature [6]. Multifocality is considered as a prognostic marker for the progression of PTC [7]. Multifocality has been associated with the high-risk features of PTC, including aggressive histology, ETE, lymph node (LN) involvement, and distant metastasis [7,8]. A recent meta-analysis also indicated that multifocality was an independent predictor of recurrence [9]. Some researchers further suggested that multifocal PTC could increase the risk of cancer-specific and overall mortality [10]. Therefore, more aggressive treatments, including total thyroidectomy and higher-dose radioiodine, are commonly used to treat patients with multifocal PTC [9,11,12,13]. 

There is a controversy about the optimal operative extent for patients with multifocal PTC. Several studies demonstrated that total thyroidectomy decreased the risk of recurrence compared with thyroid lobectomy [14,15,16]. On the contrary, other studies have suggested that lobectomy could be a feasible and valid option for patients with unilateral multifocal PTC [17,18,19,20]. These conflicting results are because, at least in part, all previous studies except one investigated the impact of operative extent without adjustment of other risk factors. Only Jeon et al. evaluated the significance of the surgical extent using a multivariable Cox proportional hazards model; however, this study only included patients with tumor size ≤ 1 cm, absence of gross ETE, and node-negative PTC (pT1aN0M0) [19]. 

Therefore, in the present study, we investigated the effect of the operative extent on the recurrence of multifocal PTC patients with various risk factors.

## 2. Materials and Methods

### 2.1. Study Design

This was a retrospective cohort study of patients aged 20 years or older with a diagnosis of unilateral multifocal PTC from 2010 to 2019 at the Ewha University Medical Center Mokdong Hospital. Institutional Review Board approval (Approval No. 2021-07-015) was obtained, and the need for written informed consent was waived.

### 2.2. Participant Selection

Patients were included in the present study if they were 20 years or older, had a pathologic diagnosis of unilateral multifocal PTC, and underwent curative-intent surgical treatment, including thyroid lobectomy or total thyroidectomy. The medical record of each patient was reviewed for clinicopathological data. Patients were excluded if they had high-risk histologic subtypes (including diffuse sclerosing, tall cell, and hobnail variants), gross ETE, or distant metastasis. Patients with incomplete data were also excluded. Data on patient demographics and tumor characteristics, including tumor size, microscopic ETE, LN metastasis, resection margin involvement, and coexisting Hashimoto thyroiditis, as well as recurrence status, were collected. The American Joint Committee on Cancer 7^th^ edition was used for pathologic tumor, node, metastasis (TNM) staging. 

### 2.3. Study End Points

The primary outcome measure was recurrence-free survival (RFS), which was defined as the time from initial surgery to the first event of recurrence. Recurrence was defined as newly found malignant lesions on the operative bed or metastatic LNs after 1 year from initial surgery, which were proven to be malignant by cytologic or histologic examination.

### 2.4. Statistical Analysis

SPSS Statistics version 23.0 (IBM Corp., Armonk, NY, USA) and R 3.5.3 (R Development Core Team, Vienna, Austria) were used for statistical analyses. Continuous data were compared by Student’s t-tests. The comparison of dichotomous data was performed by Pearson chi-squared tests. To minimize potential confounding effects and selection bias, we performed 3:1 propensity score matching [21]. We selected 4 factors that could affect the recurrence as follows: tumor size, microscopic ETE, LN metastasis, and coexisting Hashimoto thyroiditis. RFS was assessed by Kaplan–Meier survival analysis and log-rank test. As the RFS curves for lobectomy met those for total thyroidectomy, we checked the proportionality of the hazards to use a Cox proportional hazards regression model. The log(-log(survival)) plot and the scaled Schoenfeld residuals test for assessment of proportionality of hazards were used to verify the proportional hazards assumption [22]. The log(-log(survival)) plot for operative extent gave rise to reasonably parallel lines and suggested proportionality (Figure S1). The scaled Schoenfeld residuals test also produced no evidence of a poor fit (*p* = 0.42). Univariable and multivariable Cox proportional hazards regression models, therefore, were used to identify risk factors that could affect recurrence. A *p*-value < 0.05 was considered to indicate a statistically significant difference.

## 3. Results

### 3.1. Clinicopathological Characteristics of Included Patients

The baseline characteristics of 718 patients are summarized in Table 1. The mean age was 46.8 ± 11.3 years at the time of surgery, and 603 of the patients (83.6%) were women. The mean follow-up period was 5.2 ± 2.6 years. Of the 718 enrolled patients, 115 patients (16.0%) underwent ipsilateral thyroid lobectomy, and 606 patients (84.0%) underwent total thyroidectomy. Patients in the total thyroidectomy group had a larger tumor size (0.8 ± 0.5 cm vs. 0.7 ± 0.4 cm; *p* = 0.047), a higher rate of ETE (55.6% vs. 40.0%; *p* = 0.002), and an increased risk of LN metastasis (33.0% vs. 22.6%; *p* = 0.028) compared with those in the lobectomy group. Coexisting Hashimoto thyroiditis was also more common in the total thyroidectomy group than in the lobectomy group (33.7% vs. 21.7%; *p* = 0.012). Other clinicopathological factors, including age, sex, and margin involvement, showed no significant differences between the groups. 

**Figure 1:**
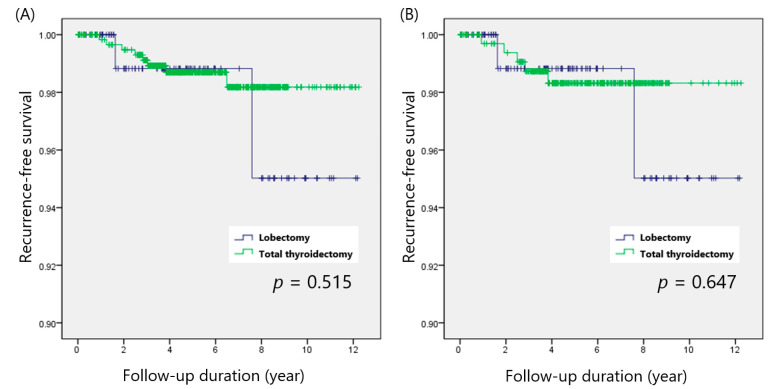
Recurrence-free survival according to the operative extent in patients with multifocal PTCs, (**A**) before and (**B**) after propensity score matching.

Recurrences were observed in 8 patients (1.3%) in the total thyroidectomy group and 2 patients (1.7%) in the lobectomy group (*p* = 0.729). In the total thyroidectomy group, 7 patients developed ipsilateral neck LN recurrences, and thyroid bed recurrence was observed in the remaining patient. All recurrences in the lobectomy group were found in the ipsilateral lateral neck LN. The log-rank test indicated that the RFS of the total thyroidectomy group (*p* = 0.515) was comparable to that of the lobectomy group (Figure 1a).

### 3.2. Recurrence-Free Survival in the 3:1 Matched Patient Group

As tumor size, microscopic ETE, or LN metastasis could affect recurrence, we performed 3:1 propensity score matching and yielded 115 matched pairs. Table 2 shows the clinicopathological comparison between the 3:1 matched total thyroidectomy and ipsilateral thyroid lobectomy groups. The matched cohorts did not differ in terms of clinicopathological features, including tumor size, microscopic ETE, and LN metastasis.

After adjusting for potential confounders, the overall recurrence rate was still comparable between the groups (1.4% vs. 1.7%; *p* = 0.826). RFS of the total thyroidectomy group (*p* = 0.647) also showed no significant difference from that of the lobectomy group (Figure 1b).

### 3.3. Predictive Factors of Poor RFS in Patients with Multifocal PTCs

The univariable Cox proportional hazards model indicated that only LN metastasis (hazards ratio [HR]: 5.370, 95% confidence interval [CI]: 1.388–20.785) was significantly associated with recurrence, and the operative extent (HR of thyroid lobectomy: 1.666, 95% CI: 0.353–7.870) was not predictive of the risk of recurrence (Table 3). LN metastasis (HR: 4.863, 95% CI: 1.179–20.056) retained statistical significance in multivariable analysis.

### 3.4. Lack of an Independent Role of Operative Extent in Patients with Other Risk Factors 

Subgroup analyses were performed to assess the impact of the operative extent in multifocal PTC patients with several risk factors. We examined 3 factors that might influence the recurrence of PTC: primary tumor size (≤1 cm or >1 cm), LN metastasis (node-negative or node-positive), and ATA risk of recurrence (low or intermediate). 

**Figure 2:**
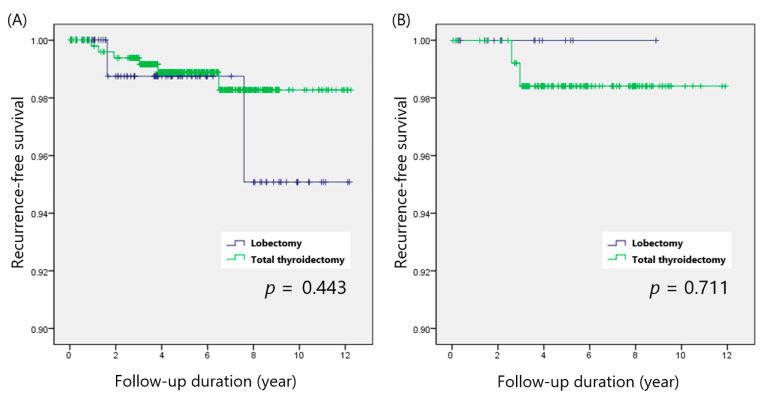
Recurrence-free survival in patients with (**A**) PTMC and (**B**) non-PTMC.

Papillary thyroid microcarcinoma (PTMC; defined as PTC ≤ 1 cm) was found in 476 patients, and 143 patients had PTC larger than 1 cm (non-PTMC). The recurrence rates of the total thyroidectomy and lobectomy groups were 6 of 476 (1.1%) and 2 of 99 (2.0%; *p* = 0.469), respectively, for PTMC patients and 2 of 127 (3.2%) and 0 of 16 (0.0%; *p* = 0.467), respectively, for non-PTMC patients. Kaplan–Meier analysis also indicated that total thyroidectomy showed comparable RFS to that of thyroid lobectomy in both the PTMC (*p* = 0.443) and non-PTMC (*p* = 0.711) groups (Figure 2A,B). 

**Figure 3:**
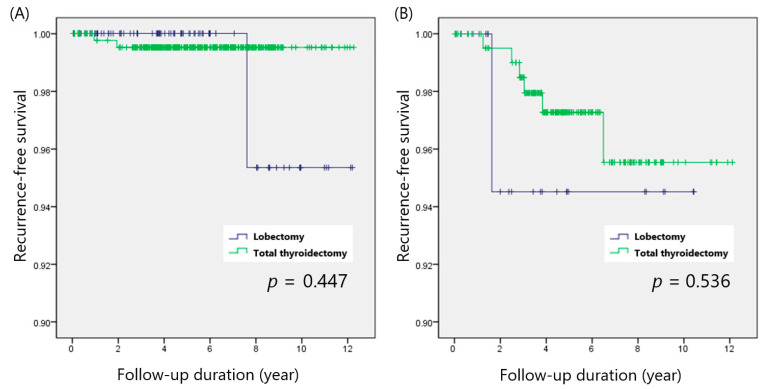
Recurrence-free survival in patients with (**A**) node-negative and (**B**) node-positive PTC.

There were 490 patients with node-negative PTC, and LN metastasis was diagnosed in 225 patients. In the node-negative group, recurrences were observed in 2 of 404 patients (0.5%) after total thyroidectomy and 1 of 89 patients (1.1%) after thyroid lobectomy (*p* = 0.490). In the node-positive group, 6 of 199 patients (3.0%) with total thyroidectomy and 1 of 26 patients (3.8%) with thyroid lobectomy developed recurrences (*p* = 0.818). RFS was not considerably different between total thyroidectomy and thyroid lobectomy in both the node-negative (*p* = 0.447) and node-positive (*p* = 0.536) groups (Figure 3A,B). 

**Figure 4:**
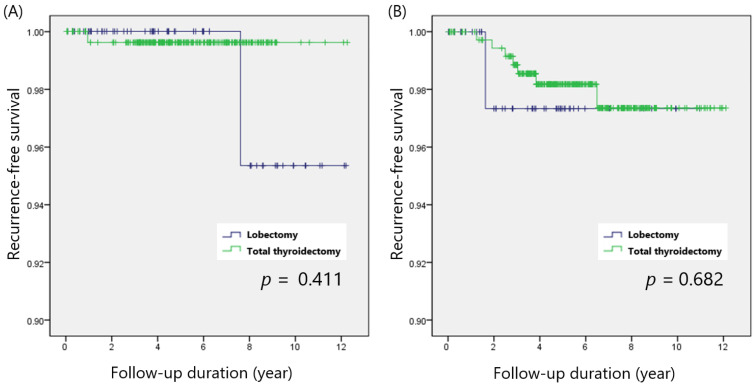
Recurrence-free survival in patients with (**A**) ATA low risk and (**B**) intermediate risk.

When we stratified patients according to the ATA risk of recurrence categories, 324 and 394 patients were classified as low risk and intermediate risk, respectively. In ATA low-risk patients, recurrence rates were comparable between the total thyroidectomy and lobectomy groups (0.4% vs. 1.5%; *p* = 0.297). Patients with ATA intermediate risk also showed no difference in the recurrence rates be-tween groups (2.0% vs. 2.0%; *p* = 0.996). Total thyroidectomy and lobectomy demonstrated similar RFS in the ATA low-risk (*p* = 0.411) and ATA intermediate-risk (*p* = 0.682) groups (Figure 4A,B). 

## 4. Discussion

The present study demonstrated that the operative extent of patients with unilateral multifocal PTC was not associated with risk of recurrence. Various guidelines and recent publications have promoted a “less is more” approach for the treatment of low-risk PTC, which represents the vast majority of thyroid cancers; this involves less extensive operation, less radioiodine, and less or no thyroid hormone suppression [23]. A global trend toward less radical surgical procedures, including thyroid lobectomy, has also gained traction in recent years [24]. Thyroid lobectomy has several advantages, including lowering the risk of complications and possibly avoiding a lifelong need for thyroid hormone supplements; however, concerns about oncological safety remain for patients with specific risk factors, including multifocality [25].

Determining the optimal surgical extent for patients with multifocal PTC has been a long-standing problem [26,27,28]. A consensus report of the European Society of Endocrine Surgeons recommended total or near-total thyroidectomy for multifocal PTC patients to reduce local recurrence [12]. A meta-analysis further indicated that patients with multifocal PTC should undergo central LN dissection [29]. On the contrary, recent studies demonstrated comparable RFS between total thyroidectomy and lobectomy for patients with node-negative multifocal PTC [18,19]. This inconsistency may be partly attributed to multifocality-associated risk factors, including large tumor size and LN metastasis, which can affect the operative extent decision [7]. These factors should also be considered to determine the optimal surgical extent for multifocal PTC.

Total thyroidectomy is usually performed instead of lobectomy for patients with more aggressive clinicopathological characteristics [30]. In the present study, compared with the thyroid lobectomy group, the total thyroidectomy group was also found to have a larger tumor size, a higher rate of microscopic ETE, and an increased risk of LN metastasis. As these high-risk features could affect the development of recurrence, propensity score matching was performed to minimize potential biases [21]. After adjusting for possible confounding factors, including tumor size, microscopic ETE, LN metastasis, and coexisting Hashimoto thyroiditis, our matched cohorts showed no difference in RFS between the total thyroidectomy and lobectomy groups. Our results further confirmed the overall oncologic safety of thyroid lobectomy for patients with multifocal PTC.

Subgroup analyses were performed to determine whether thyroid lobectomy is feasible for multifocal PTC patients with various risk factors. Total thyroidectomy showed comparable RFS to that of thyroid lobectomy for patients with non-PTMC (*p* = 0.711), node-positive PTC (*p* = 0.536), and ATA intermediate risk of recurrence (*p* = 0.682). Our findings suggest that multifocal PTC patients with tumor size > 1 cm, LN involvement, or intermediate ATA risk of recurrence do not always require a more extensive operation. Multivariable Cox proportional hazards analysis also indicated that the operative extent was not associated with the risk of recurrence, regardless of other risk factors. Therefore, we believe that thyroid lobectomy is suitable for all multifocal PTC patients without high-risk factors that require total thyroidectomy.

Our study has some limitations. First, this study was a retrospective cohort study, which is prone to selection bias. Patient selection for total thyroidectomy might be influenced by various factors, and the assignment of thyroid lobectomy and total thyroidectomy was not randomized. Although we performed propensity score matching, the results might be influenced by selection bias. Second, we did not evaluate long-term outcomes such as cancer-specific survival. During the mean follow-up period of 5.2 years, there was no cancer-specific mortality in the present study. Third, the number of patients with other risk factors of recurrence was relatively small. When we calculated the sample size for survival analysis using the data from Table 3, more than 255 recurrences were required to determine the statistical significance with HR of 1.686, 90% of power, and 2.5% of significance. Further validation studies with a larger cohort and long-term follow-up are warranted.

## 5. Conclusions

Thyroid lobectomy was not associated with the risk of recurrence in patients with multifocal PTCs. Multifocality in PTC may not always require aggressive surgery. 

## Figures and Tables

**Table 1:** Comparison of clinicopathological characteristics between lobectomy and total thyroidectomy groups.

Characteristics	Total Thyroidectomy (*n* = 603)	Thyroid Lobectomy (*n* = 115)	*p*-Value
Age (years)	47.1 ± 11.1	46.3 ± 11.1	0.489
Female sex	509 (84.4%)	91 (79.1%)	0.161
Pathologic characteristics			
Tumor size			
Mean (cm)	0.8 ± 0.5	0.7 ± 0.4	0.047
Microcarcinoma (%)	476 (78.9%)	99 (86.1%)	0.079
Microscopic ETE	335 (55.6%)	46 (40.0%)	0.002
LN metastasis	199 (33.0%)	26 (22.6%)	0.028
Margin involvement	32 (5.3%)	5 (4.3%)	0.670
Coexisting Hashimoto thyroiditis	203 (33.7%)	25 (21.7%)	0.012
Postoperative management			NA
^131^I remnant ablation	353 (58.5%)		
^131^I dose (mCi)	131.3 ± 33.3		
Follow-up period (years)	5.4 ± 2.4	4.4 ± 3.3	0.005
Recurrence	8 (1.3%)	2 (1.7%)	0.729

ETE, extrathyroidal extension; LN, lymph node; NA, not applicable.

**Table 2:** Comparison of clinicopathological characteristics between lobectomy and total thyroidectomy groups after matching.

Characteristics	Total Thyroidectomy (*n* = 345)	Thyroid Lobectomy (*n* = 115)	*p*-Value
Age (years)	46.2 ± 11.0	46.3 ± 11.1	0.938
Female sex	276 (80.0%)	91 (79.1%)	0.841
Pathologic characteristics			
Tumor size			
Mean (cm)	0.7 ± 0.5	0.7 ± 0.4	0.523
Microcarcinoma (%)	283 (82.0%)	99 (86.1%)	0.315
Microscopic ETE	136 (39.4%)	46 (40.0%)	0.912
LN metastasis	87 (25.2%)	26 (22.6%)	0.574
Margin involvement	12 (3.5%)	5 (4.3%)	0.669
Coexisting Hashimoto thyroiditis	89 (25.8%)	25 (21.7%)	0.383
Postoperative management			NA
^131^I remnant ablation	171 (49.6%)		
^131^I dose (mCi)	128.1 ± 38.6		
Follow-up period (years)	5.3 ± 2.5	4.4 ± 3.3	0.014
Recurrence	5 (1.4%)	2 (1.7%)	0.826

ETE, extrathyroidal extension; LN, lymph node; NA, not applicable.

**Table 3:** Univariable and multivariable analysis for predictive factors of recurrence in patients with multifocal PTCs.

Covariates	Univariable Analysis		Multivariable Analysis	
	HR (95% CI)	*p*-Value	HR (95% CI)	*p*-Value
Age (years)	0.958 (0.902–1.017)	0.157	0.958 (0.899–1.021)	0.189
Male sex	2.289 (0.592–8.854)	0.230	1.637 (0.403–6.641)	0.490
Tumor size (cm)	1.027 (0.349–3.022)	0.962	0.805 (0.192–3.377)	0.767
Microscopic ETE	1.270 (0.358–4.503)	0.711	1.137 (0.290–4.457)	0.853
LN metastasis	5.370 (1.388–20.785)	0.015	4.863 (1.179–20.056)	0.029
Margin involvement	0.046 (0.000–6959.675)	0.612	0.000 (0.000-infinite)	0.982
Hashimoto thyroiditis	0.253 (0.032–2.002)	0.193	0.288 (0.035–2.402)	0.250
Operative extent	1.666 (0.353–7.870)	0.519	1.686 (0.321–8.852)	0.537

HR, hazard ratio; CI, confidence interval; ETE, extrathyroidal extension; LN, lymph node.

## Data Availability

The data presented in this study are available on request from the corresponding author. The data are not publicly available due to institutional policy.

## Supplementary Materials

The following is available online at https://www.mdpi.com/article/10.3390/cancers14020432/s1: Figure S1. Comparison of survival analysis by the operative extent: log(-log(survival)) plot.
