# Supplementary figures and images for: Optimal Surgical Extent in Patients with Unilateral Multifocal Papillary Thyroid Carcinoma
Source: Cancers (Basel). 2022 Jan 15;14(2):432. doi: 10.3390/cancers14020432 (PMC8773701; doi:10.3390/cancers14020432)

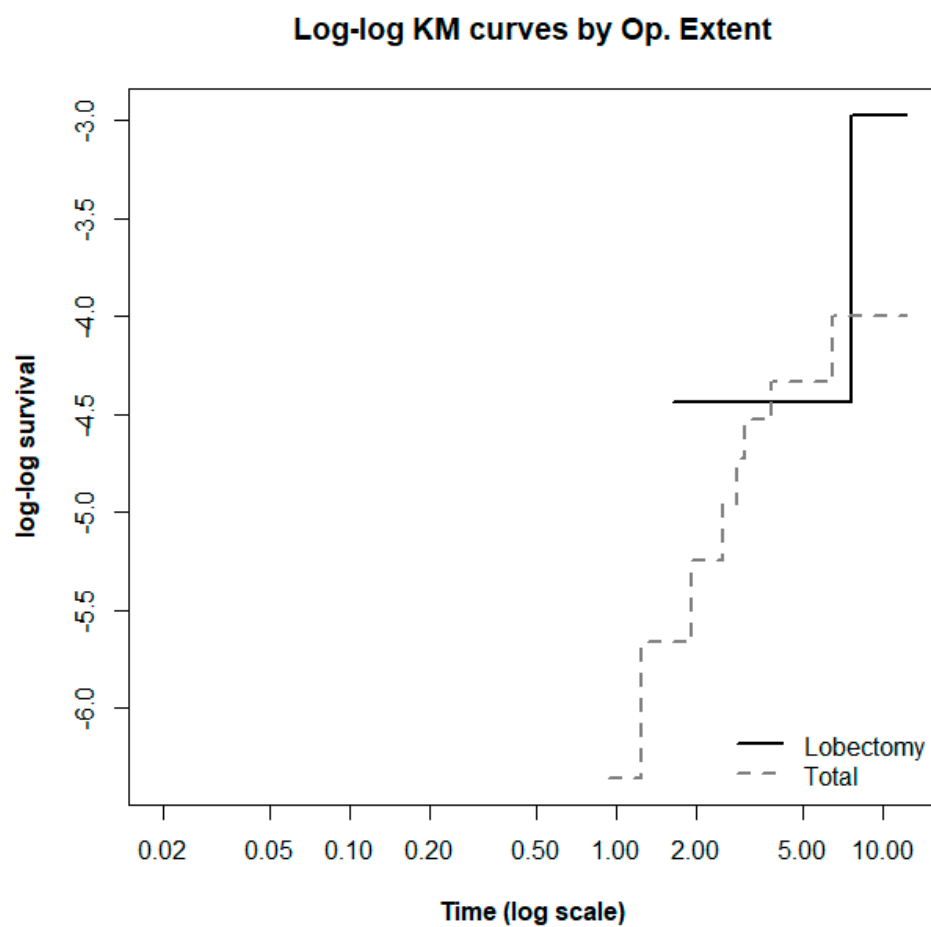

**Figure S1.** Comparison of survival analysis by the operative extent: log(-log(survival)) plot.

Supplement: Supplementary file 1 [file cancers-14-00432-s001.zip › cancers-1529895-supplementary.pdf]
